# Investigating Alterations of Social Interaction in Psychiatric Disorders with Dual Interactive Eye Tracking and Virtual Faces

**DOI:** 10.3389/fnhum.2014.00758

**Published:** 2014-09-23

**Authors:** Bert Timmermans, Leonhard Schilbach

**Affiliations:** ^1^Social Interaction and Consciousness Lab (SINC), School of Psychology, University of Aberdeen, Aberdeen, UK; ^2^Neuroimaging Group, Psychiatry and Psychotherapy Clinic, University Hospital of Cologne, Cologne, Germany

**Keywords:** eye tracking, social interaction, anthropomorphic avatars, autism, schizophrenia

## Psychiatric Disorders as Disorders of Social Interaction

Impairments of social interaction and communication are an important if not essential component of many psychiatric disorders. In the context of psychopathology, one tends to think predominantly of autism spectrum disorders. However, many psychopathologies are to some degree characterized by alterations or impairments of interpersonal functioning in the DSM-5 (American Psychiatric Association, 2013), for instance schizophrenia [even auditory hallucinations have been linked to social cognition; (Bell, 2013)], or personality disorders such as borderline personality disorder (Wright et al., 2013). For different pathologies, the difficulties in social interaction may originate in different impairments; for instance in schizophrenia they may be related to a deficit in context processing (Cohen et al., 1999). Still, irrespective of the specific place that social interaction impairments take within different etiologies, it is clear that the systematic study of interaction patterns could teach us a lot about how they manifest themselves in patients, how healthy people with whom the patients interact engage with these patterns, and how they relate to underlying neurobiology. Here, we argue why this should and how this could be accomplished.

One important aspect of social interaction that is increasingly shown to be impaired in psychiatric disorders is the recognition and production gaze behavior, often related to disorder-specific attentional bias (Armstrong and Olatunji, 2012). Schizophrenia has been associated with gaze-related attention deficits (Tso et al., 2012; Dalmaso et al., 2013). A recent study shows that patients with schizophrenia can be distinguished from neurotypical controls with astonishing accuracy on the basis of abnormal eye-tracking patterns on simple tasks such as fixation and smooth pursuit (Benson et al., 2012). Depression and bipolar disorder have been associated with prefrontal and cerebellar disturbances of oculomotor control during episodes of major depression, problems with antisaccade tasks (production of saccades away from a cue), and delayed initiation of saccades made on command (Sweeney et al., 1998). Finally, it is well known that people with autism orient to different kinds of contingencies (Gergely, 2001; Klin et al., 2009).

**Figure 1:**
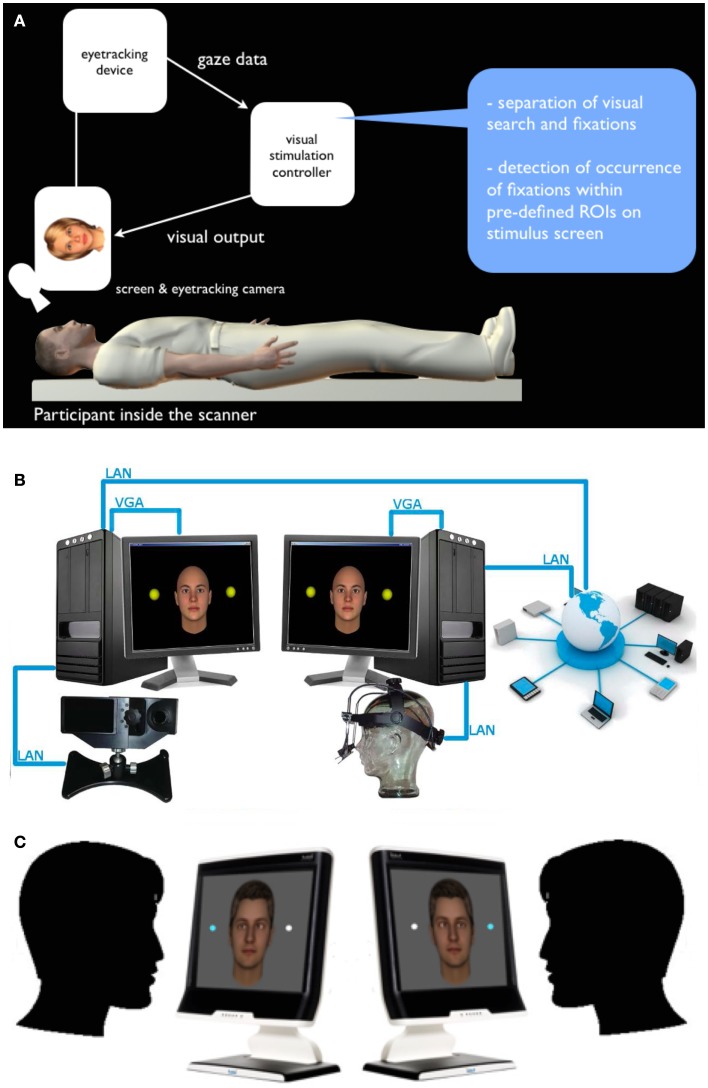
**Interactive and dual eye-tracking with virtual anthropo- morphic avatars**. **(A)** Interactive eye-tracking setup operationalized for fMRI: a virtual character is shown on screen and can be made “responsive” to the participant’s looking behavior by means of an algorithm-based, real-time analysis of the eye-tracking obtained from the study participant. **(B)** Schematic setup: two eye-tracking devices are linked via a local area network (LAN), which allows to simultaneously measure two study participants engaged in a mediated gaze-based interaction (each participant is represented by a virtual character for the respective other). **(C)** Two participants engaged in a two-person perceptual decision-making task, in which both are asked to discriminate stimuli while the gaze behavior of the respective other participant is visualized on the stimulus screen as well. Importantly, people do not just see where the other is looking via cursor or similar, but actually experience the other’s gaze, to which they can dynamically adapt.

However, most of the experimental paradigms used to establish gaze anomalies are essentially non-interactive and focus on how particular clinical populations differentially perceive stimuli or social scenes, passively. Likewise, the study of social cognition has only in the last decade begun to incorporate social interaction into its explanation of how we come to understand others and how we manage to navigate a complex social world (Schilbach et al., 2013). This “interactive turn” marks a departure from more traditional approaches, which have emphasized the importance of being able to think about the mental states of others. We have argued that the core problem with social interaction in clinical populations may not only lie in passive perception of social cues, but rather in a skewed experience of how one’s own actions influence the social world and in patient’s abilities to automatically and rapidly generate behavioral adjustments in response to social stimuli (Schilbach et al., 2012, 2013). Recently, methodological advances have allowed for the study of real-time dynamic social coordination in for instance children with autism (Fitzpatrick et al., 2013), but while undoubtedly rich, the problem with full-body social interaction is precisely that it is so rich, which makes it most difficult to operationalize so as to be used to quantify aspects of interpersonal coordination. Furthermore, fully interactive approaches become problematic if one wanted to use neuroimaging techniques that can access deeper brain structures, such as fMRI, to investigate the neural correlates of interpersonal coordination in on-going social interactions. Indeed, we have showed that the experience of self-initiated (gaze-based) contingencies is linked to activity in the brain’s reward system, notably the ventral striatum [Pfeiffer et al. (2014); Figure 1A], and it has been suggested that for instance individuals with autism may have difficulties with precisely those rewarding aspects of social interaction (Schmitz et al., 2008; Kohls et al., 2012).

## Gaze and Avatars to Study Real-Time Social Interaction

Interactive and even dual interactive eye tracking have been around for a couple of years (Richardson and Dale, 2005; Sangin et al., 2008; Carletta et al., 2010; Neider et al., 2010). Interactive eye tracking is a method whereby a person’s eye gaze is tracked and fed back into the on-going experiment, not so much as a behavioral response akin to a button press but rather as a way of making the trial or experiment course in some way contingent upon the person’s gaze. In dual interactive eye tracking, the gaze of two participants is simultaneously tracked and not only fed into their own experimental course, but also in that of the other person. Due to different experimental questions, all dual eye-tracking setups have either simply collected joint gaze data (non-interactive), or used them to display for one person where the other was looking or reading, by means of a pointer or a little rectangle. While we do not deny the merits of these methods, in social interaction one does not see where others are looking via a rectangle overlaid on a scene (though probably with Google Glass this is not so far away). Instead, what is minimally needed to emulate social interaction is the *visibility of one person’s social cue to the other*. One logical option is to have people watch live videos of one another (Redcay et al., 2010), but the disadvantage of this is that facial features provide massive social cues that are not always controllable, and that live videos only allow for delay of the video or playing back an unrelated recorded sequence, but do not allow a systematic manipulation of interaction contingencies.

In order to combine both the experimental controllability of depicting the other’s gaze via an on-screen stimulus and the social aspect of perceived gaze, we developed a setup in which a person’s eye gaze either influences an avatar’s gaze behavior [simple interactive; (Wilms et al., 2010; Pfeiffer et al., 2011); Figure 1A], or is displayed onto the eyes of the avatar on another person’s screen and vice versa [dual interactive; (Barišic et al., 2013); Figure 1B]. It has been shown that virtual avatars can robustly elicit social effects comparable to real faces, for instance, social inhibition and facilitation, interpersonal distance regulation and social presence, empathy, and pro-social behavior have been shown to be comparable with virtual avatars (Bailenson et al., 2003; Hoyt et al., 2003; Bente et al., 2007; Gillath et al., 2008; Slater et al., 2009). Therefore, using anthropomorphic virtual characters and making them interactive provides an excellent compromise of ecological validity and experimental control. Using the dual eye-tracking setup, in particular, one can generate two-person tasks, during which an integration of the interaction partner’s gaze behavior may (or may not) become relevant for task performance and measures of subjective experience (Figure 1C).

## Empirical Questions and Pathologies

We see four ways in which interactive and dual setups as described above can be useful for psychiatry. First, a simple interactive eye-tracking setup, which allows for control of the algorithm by which the avatar behaves in response to the person’s gaze, could be used for diagnosis just as the setup used by Benson and colleagues (Benson et al., 2012), which had people perform three simple tasks: smooth pursuit, a fixation stability task, and a free-viewing tasks, but more along a social dimension, in that it would tell us to what degree persons are sensitive to action contingencies, or the disruption thereof.

Second, dual interactive setups would allow us to start looking at whether and how particular psychopathologies are associated with skewed interaction patterns. Indeed, the major advantage of a dual interactive setup is that it allows for a precise quantification of the gaze-interaction dynamics, using non-linear methods such as cross-recurrence quantification analysis. Such quantified interaction dynamics have been shown to correlate with person perception (Miles et al., 2009) and social motives (Lumsden et al., 2012), and have shown a deficit in simultaneous movement synchronization in children with Autism Spectrum Disorder (Fitzpatrick et al., 2013). Thus, it would be possible to tease apart the degree to which patients (a) elicit gaze patterns that differ from controls (and entrain controls), (b) are differentially sensitive to controls’ gaze patterns, (c) are differentially sensitive to how their gaze impacts a control person’s and vice versa, and (d) are differentially sensitive to the communicative signals that certain variance in the other’s gaze or in the dyadic gaze patterns entails. Establishing such measures would lend itself to neuroimaging purposes, which could investigate the neural correlates of social interaction dynamics in one or both brains of the interaction partners. Also, the fact that the setup is virtual means that it is possible to manipulate this virtual environment in such a way that interactors perceive different scenes and one can study the degree to which communication breaks down in certain cases.

Third, a dual setup would allow for quantification not simply of a clinically significant aberration in gaze pattern, but rather of the diagnostic intuition: what does a clinician do and how does the patient have to react in order to be diagnosed as belonging to a particular clinical group?

Finally, a dynamically interactive setup could be implemented therapeutically. For instance, the currently existing VIGART system [virtual interactive system with gaze-sensitive adaptive-response technology, (Lahiri et al., 2011a,b)] has participants interact with a virtual avatar while their gaze is monitored in real-time. Following the interaction, participants receive feedback about their gaze behavior, which helps adolescents with Autism Spectrum Disorder improve their eye gaze patterns. A fully dual interactive eye-tracking setup would allow such feedback in real time via calculated indices not just of gaze behavior but of gaze contingencies.

Thus, just as from a research point of view dual setups will allow us to study social cognition in a truly social setting, such setups, particularly when implemented with eye tracking and virtual avatars, would allow us to look at psychopathology in terms of the clinical symptoms being embedded (and perhaps reinforced) by the social environment, as both try and engage in a social interaction for which each has a different “sketchbook.” Indeed, persons with autism often report problems in interaction with non-autistic persons, but not so much with other persons with autism. Such questions can only be addressed in interactive setups, whereby the use of virtual avatars provides many advantages.

## Conflict of Interest Statement

The authors declare that the research was conducted in the absence of any commercial or financial relationships that could be construed as a potential conflict of interest.
